# Intravitreal Vascular Endothelial Growth Factor Inhibitor Therapy in Denmark and 5-Year Projections

**DOI:** 10.1001/jamanetworkopen.2023.35148

**Published:** 2023-09-22

**Authors:** Benjamin Sommer Thinggaard, Frederik Pedersen, Jakob Grauslund, Lonny Stokholm

**Affiliations:** 1 Department of Clinical Research, University of Southern Denmark, Odense; 2 Department of Ophthalmology, Odense University Hospital, Odense, Denmark; 3 Open Patient Data Exploratory Network (OPEN), Odense University Hospital, Odense, Denmark

## Abstract

This cohort study examines the use of intravitreal injections of vascular endothelial growth factor inhibitor in Denmark from 2007 through 2022 and forecasts future demand for this therapy.

## Introduction

The introduction of intravitreal vascular endothelial growth factor (VEGF) inhibitor therapy has revolutionized treatment for neovascular age-related macular degeneration (nAMD), retinal vein occlusion (RVO), and diabetic macular edema (DME) and halved the number of people aged 50 years or older newly diagnosed with blindness.^1^ The number of patients receiving VEGF inhibitors is expected to escalate as life expectancy increases,^2,3^ influencing strategic planning for future ophthalmological services. Thus, we assessed the current use of VEGF inhibitors and forecast the growing demand for this therapy in Denmark.

## Methods

This descriptive nationwide cohort study used data from the Danish National Patient Register and the Danish Civil Registration System. Data obtained included birth date, diagnosis codes, and procedure codes for 4 059 802 individuals older than 40 years between January 1, 2007, and December 31, 2022. In accordance with Danish Health Data Authority policy, this study was exempt from review and informed consent, as it was registry-based research. We followed the STROBE reporting guideline.

Patients with nAMD, RVO, or DME were identified in the Danish National Patient Register using *International Statistical Classification of Diseases and Related Health Problems, Tenth Revision (ICD-10)* codes for first diagnosis of nAMD (H353, H353J, H353K), RVO (H348*), or DME (H360*) in combination with at least 1 injection of intravitreal VEGF inhibitors (Nordic Medico-Statistical Committee Classification of Surgical Procedures code KCKD05B). We calculated the 5-year forecast for these injections as linear growth based on data from 2017 to 2022 (eMethods in Supplement 1).

## Results

Between 2007 and 2022, 901 826 injections with VEGF inhibitors were administered to 56 081 Danish patients (median [IQR] age at initial injection, 77.2 [69.7-83.5] years; 32 505 women [58.0%] and 23 576 men [42.0%]). The overall use rate was 13.8 injections per 1000 patients, including 9.2 injections per 1000 patients with nAMD, 2.0 injections per 1000 patients with RVO, and 1.6 injections per 1000 patients with DME. No diagnosis could be identified for 4789 patients (8.5%).

There was an annual increase of more than 10 000 injections from 2019 onward, with 95 677 injections in 2019 and 131 010 injections in 2022 (Table). Although the annual incidence of patients with nAMD, RVO, or DME receiving VEGF inhibitor therapy remained consistent between 2019 and 2022, the overall number of injections for nAMD increased from 12 992 in 2019 to 17 338 in 2022.

**Table.** Vascular Endothelial Growth Factor (VEGF) Inhibitor Therapy From 2007 to 2022 Stratified by Underlying Diagnosis

Diagnosis	No. of patients	No. of VEGF inhibitor injections		
		Total	2019	2022
Total	56 081	901 826	95 677	131 010
nAMD	37 186	696 055	73 836	99 680
RVO	7795	86 161	9821	15 069
DME	6351	70 104	7072	8428
Other^a^	4789	49 506	4948	7833

Abbreviations: DME, diabetic macular edema; nAMD, neovascular age-related macular degeneration; RVO, retinal vein occlusion.

^a^ Other indicates unknown diagnosis.

Extrapolating data from 2017 to 2022, we project a 50% increase in the number of VEGF injections for nAMD, a 56% increase for injections for RVO, and a 24% increase in injections for DME in 2027. This represents an overall 50% increase in the total number of injections (Figure).

**Figure.**
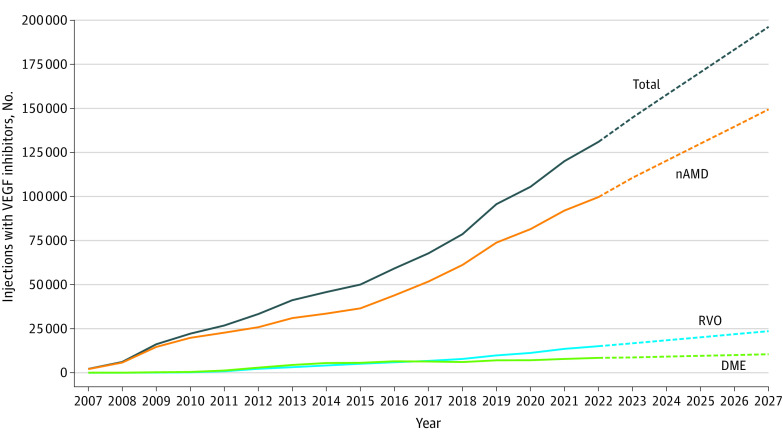
Vascular Endothelial Growth Factor Inhibitor Therapy in Denmark From 2007-2022 and 5-Year Growth Projections Dashed lines represent projections through 2027. DME indicates diabetic macular edema; nAMD, neovascular age-related macular degeneration; and RVO, retinal vein occlusion.

## Discussion

Based on our forecast, 50% more injections with VEGF inhibitors will be needed in Denmark in 5 years. We project an equal increase in the number of injections in patients with RVO and nAMD, given their association with increasing age. A minor increase in injections in patients with DME is expected, given improvements in antidiabetic treatment and early detection of DME during the past 10 years due to the increase in patients with diabetes.^4,5^ A recent study^6^ of patients with nAMD revealed a projected linear growth of the number of patients with nAMD requiring VEGF inhibitors. These results imply that the demographic change (ie, increasing age) might turn into the main factor contributing to growth.

A study limitation is that the underlying diagnosis prompting treatment with VEGF inhibitors could not be determined for 8.5% of the patients, yet this uncertainty does not impact our 5-year projections. Future development of alternative methods for delivering VEGF inhibitors, such as refillable ports, depots, and implants, could potentially reduce the frequency of individual injections. Still, the forecasted increase in VEGF inhibitor injections based on these nationwide data might facilitate accurate planning of future ophthalmological services.

## Notes

Supplement 1.
eMethods.

Supplement 2.
Data Sharing Statement
