# Error in the Results

**DOI:** 10.1001/jamanetworkopen.2023.40286

**Published:** 2023-10-11

In the Research Letter, “Intravitreal Vascular Endothelial Growth Factor Inhibitor Therapy in Denmark and 5-Year Projections”^1^ published online September 22, 2023, the second sentence of the Results section is incorrect. The sentence should be the following: “The overall median use rate was 9 injections per patient, including 11 injections per patient with nAMD [neovascular age-related macular degeneration], 7 injections per patient with RVO [retinal vein occulsion], and 6 injections per patient with DME [diabetic macular edema].” This article has been corrected.
