# Supplementary material for: Intravitreal Vascular Endothelial Growth Factor Inhibitor Therapy in Denmark and 5-Year Projections
Source: JAMA Netw Open. 2023 Sep 22;6(9):e2335148. doi: 10.1001/jamanetworkopen.2023.35148 (PMC10517372; doi:10.1001/jamanetworkopen.2023.35148)
Supplement: Supplement 1. — eMethods. [file jamanetwopen-e2335148-s001.pdf]

Supplemental Online Content

Thinggaard BS, Pedersen F, Granuslund J, Stokholm L. Intravitreal vascular endothelial growth factor inhibitor therapy in Denmark and 5-year projections. *JAMA Netw Open*. 2023;6(9):e2335148. doi:10.1001/jamanetworkopen.2023.35148

eMethods

This supplemental material has been provided by the authors to give readers additional information about their work.

© 2023 Thinggaard BS, et al. *JAMA Network Open.*

eMethods

Some patients had more than one disease registered (nAMD, RVO or DME). For these patients, we registered the disease that was identified in the timeframe of two years before or after the first injection. Patients with more than one disease within this timeframe were labeled as *other* in Table 1 as were patients with no diagnostic code.

A prespecified significance level of 5% (corresponding to 95% confidence intervals) was utilized and all hypothesis tests and confidence intervals were two-sided. We used Stata 17.0 for analysis (StataCorp LLC. College Station, TX, USA).

The study was assigned a record number (FSEID-00004087) by the Danish Health Authorities for the extraction and processing of data. Relevant permissions were obtained from the Region of Southern Denmark's record of data processing activities (journal no 19/7775). However, ethical approval is not required for registry-based research conducted in Denmark.

© 2023 Thinggaard BS, et al. *JAMA Network Open.*
