# Supplementary material for: Intravitreal Vascular Endothelial Growth Factor Inhibitor Therapy in Denmark and 5-Year Projections
Source: JAMA Netw Open. 2023 Sep 22;6(9):e2335148. doi: 10.1001/jamanetworkopen.2023.35148 (PMC10517372; doi:10.1001/jamanetworkopen.2023.35148)
Supplement: Supplement 2. — Data Sharing Statement [file jamanetwopen-e2335148-s002.pdf]

# Data Sharing Statement

Thinggaard. Intravitreal Vascular Endothelial Growth Factor Inhibitor Therapy in Denmark and 5-Year Projections. *JAMA Netw Open*. Published September 22, 2023. doi:10.1001/jamanetworkopen.2023.35148

**Data**
**Data available:** No
